# Dual sources of water overprinting on the low zircon δ^18^O metamorphic country rocks: Disequilibrium constrained through inverse modelling of partial reequilibration

**DOI:** 10.1038/srep40334

**Published:** 2017-01-16

**Authors:** Chun-Sheng Wei, Zi-Fu Zhao

**Affiliations:** 1 CAS Key Laboratory of Crust-Mantle Materials and Environments, School of Earth and Space Sciences, University of Science and Technology of China, Hefei 230026, China

## Abstract

Since water is only composed of oxygen and hydrogen, δ^18^O and δ^2^H values are thus utilized to trace the origin of water(s) and quantify the water-rock interactions. While Triassic high pressure (HP) and ultrahigh pressure (UHP) metamorphic rocks across the Dabie-Sulu orogen in central-eastern China have been well documented, postcollisional magmatism driven hydrothermal systems are little known. Here we show that two sources of externally derived water interactions were revealed by oxygen isotopes for the gneissic country rocks intruded by the early Cretaceous postcollisional granitoids. Inverse modellings indicate that the degree of disequilibrium (doD) of meteoric water interactions was more evident than that of magmatic one (−65 ± 1^o^ vs. −20 ± 2°); the partial reequilibration between quartz and alkali feldspar oxygen isotopes with magmatic water was achieved at 340 °C with a water/rock (W/R) ratio of about 1.2 for an open-hydrothermal system; two-stage meteoric water interactions were unraveled with reequilibration temperatures less than 300 °C and W/R ratios around 0.4. The lifetime of fossil magmatic hydrothermal system overprinted on the low zircon δ^18^O orthogneissic country rocks was estimated to maintain up to 50 thousand years (Kyr) through oxygen exchange modellings. Four-stage isotopic evolutions were proposed for the magmatic water interacted gneiss.

Water-rock interactions accompanying with magmatism and metamorphism were globally documented^1^,^2^,^3^,^4^,^5^,^6^,^7^,^8^,^9^. For magmatism (in particular plutonism) driven hydrothermal systems, the intrusives and their country rocks interacted with waters were characterized through stable isotopes^10^,^11^,^12^,^13^,^14^,^15^. However, there still exist uncertainties such as: (1) which mainly contributed to interact with rocks, externally infiltrated or internally buffered water, or both; (2) whether igneous and country rock were interacted with the commonly originated water, or they were interacted with different sources of water; (3) what were the operated processes of water-rock interaction, they were closed- or open-hydrothermal systems; and (4) what were the temperature, W/R ratio and duration for the water-rock interactions. In order to address these issues, the early Cretaceous postcollisional granitoids and their country rocks of Triassic gneisses from the Dabie orogen in central-eastern China were studied.

The Dabie-Sulu orogen is characterized by the largest occurrence of the microdiamond- and/or coesite-bearing UHP metamorphic rocks worldwide^16^,^17^,^18^,^19^,^20^. Triassic ages of 200 to 240 Ma were dated with distinctive geochronometers for the eclogite facies rocks and their exhumation cooling histories were accordingly quantified^21^,^22^,^23^,^24^,^25^,^26^,^27^,^28^. Furthermore, the world-record ultrahigh ε_Nd_(t) value up to +264 was measured for eclogites^29^ and zircons with the ever reported lowest δ^18^O values down to about −11‰ were found^30^,^31^.

In contrast to the sporadically outcropped lenses or blocks of UHP eclogites, composite plutons and batholiths of granitoid are predominant igneous rocks although a number of coeval small mafic to ultramafic plutons are documented in the Dabie orogen. The studied granitoids and their gneissic country rocks of the Tianzhushan pluton (TZS) is adjacent to the Central Dabie UHP metamorphic belt of the Dabie Block (DBB and refer to Fig. 1 in refs 32, 33 and especially Fig. 5 in ref. 34). The intrusive contact between granitoids and gneisses was unambiguously observed in the field. For comparison, two gneisses from the Sidaohe without intruding granitoids in the Hong’an Block were also studied.

Fresh medium-grained granitoids and gneisses were collected from quarries and/or along road cuttings. Petrographically, quartz, feldspar, biotite, and sometimes amphibole are major rock-forming minerals, and accessory minerals include zircon and magnetite. Zircon δ^18^O values with quartz and alkali feldspar data from the same set of samples were analyzed via laser fluorination online techniques (Methods and Table S1).

## Results

**Figure 1:**
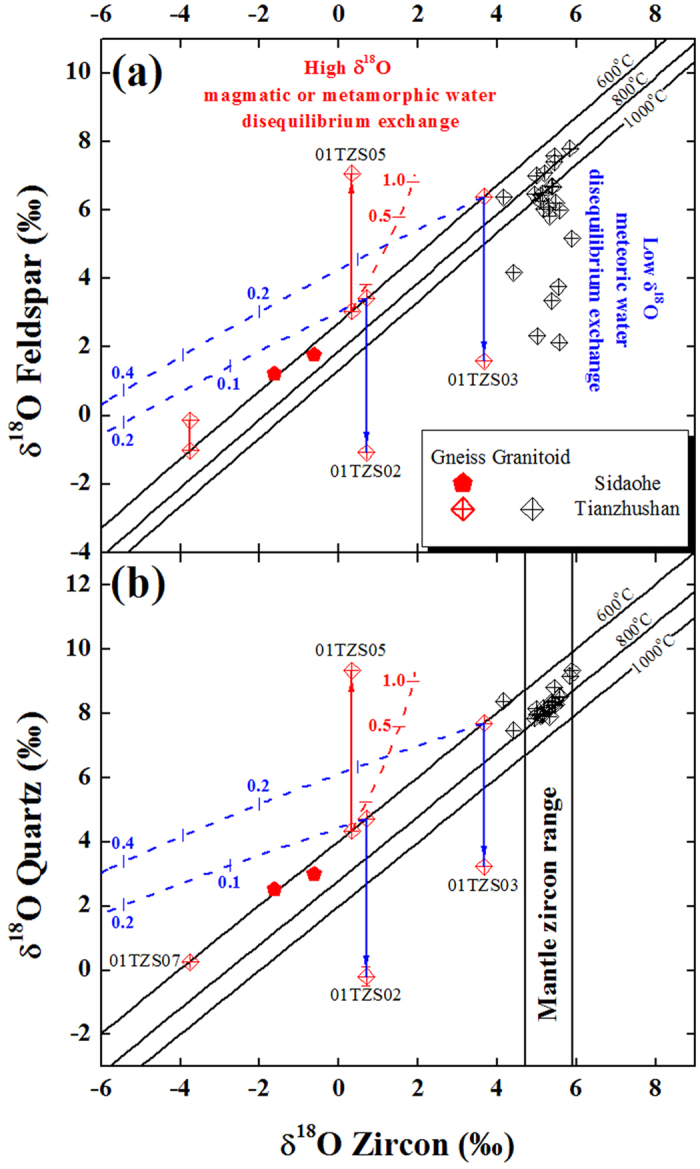
Diagrams of zircon versus alkali feldspar (**a**) and quartz δ^18^O values (**b**) for the granitoids and their gneissic country rocks in the Dabie orogen. Except for few data points, the error bar for most samples is smaller than the symbol size. Solid lines are calculated isotherms^35^, and two vertical solid lines in (**b**) denote the mantle zircon δ^18^O value of 5.3 ± 0.6‰ for comparison^36^. Arrowed solid lines denote the observed disequilibrium oxygen isotopes, and dashed curves are open-system modellings of magmatic (red) and meteoric water interactions (blue), respectively. Small ticks with numbers are W/R ratios. Data and parameters refer to Tables S1 and S2.

As shown in Fig. 1, zircon δ^18^O values of the gneisses scatter from −3.78 to 3.67‰, whereas zircon values of granitoids cluster around 5.32 ± 0.34‰ (1 SD, n = 23) and overlap with mantle zircon. This suggests that the granitoids with uniform zircon δ^18^O values cannot petrogenetically link with these low zircon δ^18^O heterogeneous orthogneisses.

On the other hand, some of the alkali feldspar δ^18^O values of granitoids steeply shift toward low values and depart from equilibrium isotherms, indicating low δ^18^O water interactions. Both lowered and elevated alkali feldspar δ^18^O values, however, were observed for the gneissic country rocks of the Tianzhushan pluton, suggesting that at least two sources of water were interacted with them on the pluton scale. Furthermore, it is of particular interests that the remarkable disequilibria and even reversals are evident not only between zircon and alkali feldspar but also between zircon and quartz δ^18^O values for these gneissic country rocks.

One of gneissic country rocks around the Tianzhushan granitoid pluton, however, still retained equilibrium oxygen isotope fractionation between zircon and quartz (labelled 01TZS07 in Fig. 1b), and about 600 °C of equilibrium temperature (Teq) was accordingly calculated. Because this temperature is within the ranges from 585 to 655 °C for the two gneisses unexperienced water-rock interactions from the Sidaohe, it is thus of geologic significance and applied to estimate the initial quartz and alkali feldspar δ^18^O values in the following water interaction modellings.

## Discussion

Because oxygen diffusivity of zircon is considerably slower than that of quartz and alkali feldspar (Fig. S1a), zircon is thus one of the most resistant accessory minerals to water interactions and can somehow maintain its original δ^18^O value. Disequilibria of oxygen isotopes accordingly occur among the resistant and susceptible minerals during short-lived hydrothermal processes (Fig. 1), in good agreement with their kinetic behaviors. Since the discrepancy of oxygen diffusion rates between quartz and alkali feldspar is less pronounced (Fig. S1a), partial reequilibrations of common rock-forming minerals were achieved for some of gneissic country rocks in the course of water-rock interactions. These enable the following modellings possible and reliable.

### Modelling of high δ^18^O water interactions

Because alkali feldspar and/or quartz δ^18^O values were elevated and departed from isotherms for samples 01TZS05 and 01TZS07 (Fig. 1), high δ^18^O water interactions were modelled to reproduce the observed variations.

**Figure 2:**
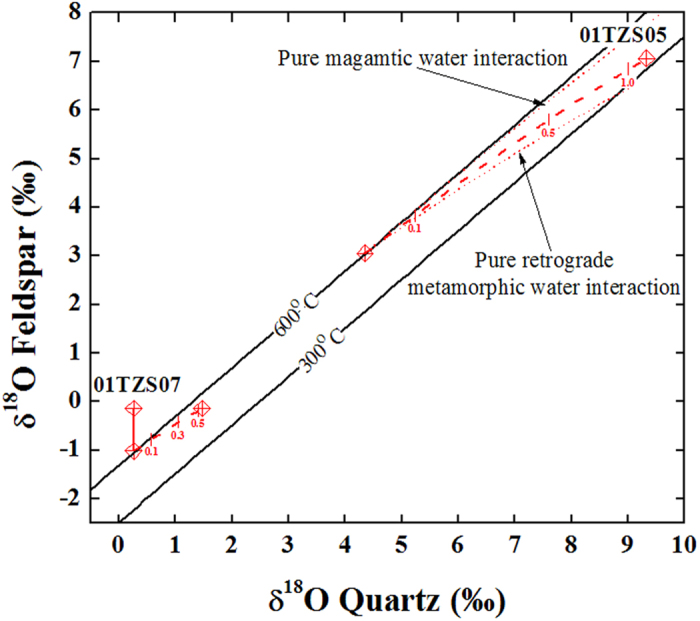
Diagram of quartz versus alkali feldspar δ^18^O values for the gneissic country rocks interacted with high δ^18^O water in the Dabie orogen. Dashed curves are inverse modellings of open-systems. Dotted curves are forward modellings with pure magmatic and retrograde metamorphic waters, respectively, which were calculated with zircon δ^18^O and Teq values of the Tianzhushan granitoids and gneissic country rock. Others refer to Fig. 1.

It can be seen in Fig. 2 that quartz oxygen isotope was reequilibrated with alkali feldspar at 340 °C for sample 01TZS05. In terms of inverse procedures described in the Methods (Eqs. (1) through (3), 7, 8), the initial δ^18^O value of magmatic water was constrained as 4.21‰ for an open-system. Based on parameters listed in Table S2, the observed δ^18^O values were successfully modelled (Fig. S2 and Fig. 2). A minimum W/R ratio of about 1.2 was inferred in this case. In fact, this W/R ratio was the time integrated value over the lifetime of the hydrothermal activity and the actual W/R ratio could be even larger. Nevertheless, the W/R ratio up to 1.2 for sample 01TZS05 probably implied that magmatic water was intensively channelized flow outward from the pluton into the gneissic country rock and hydrostatically controlled by fractures and/or fissures.

Compared to the forward modelling with pure magmatic water (labelled dotted curve in Fig. 2), the inversed initial δ^18^O value of magmatic water was somehow lowered for sample 01TZS05 (Table S2). There are two kinds of hypothetical pathways to account for this scenario, i.e., magmatic water cooling vs. meteoric water mixing. In order to reproduce the required δ^18^O value, the magmatic water had to diabatically cool down to about 175 °C (line 1 in Fig. S3), which was too low to model the observed values. On the other hand, there are three subsets of pathways for the involvement of low δ^18^O meteoric water. Among them, the isothermal binary mixing between cooled magmatic and heated meteoric water under condition of 340 °C is more realistic (line 2-3 in Fig. S3). Moreover, the forward modelling with pure retrograde metamorphic water cannot reproduce the observed values, either (labelled dotted curve in Fig. 2). While the mixing between magmatic and retrograde metamorphic waters can equivalently account for the initial δ^18^O value of required magmatic water, it was geochronologically less likely. The metamorphism was at least 100 million years earlier than magmatism in the Dabie orogen, they were distinct geological events and their fluids thus cannot be simultaneously mixed each other. In this case, the gneissic country rock of sample 01TZS05 was interacted with externally derived fluids, which was driven by the early Cretaceous postcollisional magmatism.

Based on Eq. (4), data and parameters listed in Tables S1, S2 and modelling results in Fig. S2, the doD value was calculated ranging from −22 to −18° (averaged −20 ± 2°) for sample 01TZS05. It is worthwhile pointing out that the minus symbol of doD value denoted anticlockwise rotations for the observed δ^18^O values relative to thermodynamic equilibrium exchanged values due to zircon being more resistant to water interactions (red solid vs. dashed lines in Fig. 1).

Due to disequilibrium between quartz and alkali feldspar oxygen isotopes for sample 01TZS07 (Fig. 2), an apparent temperature higher than Teq value of quartz-zircon pair was yielded. Because this observed disequilibrium was resulted from the preferentially elevated δ^18^O value of alkali feldspar, interactions with either magmatic or metamorphic water were thus inferred. On the other hand, under reequilibration conditions, a relationship of 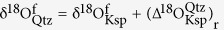 is theoretically held. Substituting this into left-hand term of Eq. (2) and combining it with Eq. (1), the hypothetical reequilibration temperature can be inversed. Based on the observed alkali feldspar δ^18^O value and initial oxygen isotopes of quartz and alkali feldspar (Tables S1 and 2), a reequilibration temperature of about 490 °C was solved with the internally buffered retrograde metamorphic water δ^18^O value of −0.86‰. It is of interests to point out that this reequilibration temperature was inversed through open-system model, an unrealistic value up to 830 °C was yielded if closed-system model was adopted. Moreover, unreasonable reequilibration temperatures over 600 °C were yielded if magmatic waters were assumed. It can be seen in Figs. S2 and 2 that the observed alkali feldspar δ^18^O value of sample 01TZS07 can be well modelled with a W/R ratio of about 0.55 for an open-system. Due to the kinetic resistance to water interaction for quartz, only the susceptible alkali feldspar was reequilibrated with the internally buffered retrograde metamorphic water during exhumation process. Under this circumstance, a disequilibrium between quartz and alkali feldspar oxygen isotopes for sample 01TZS07 was thus resulted in and the corresponding doD value of −54° was according yielded.

### Modelling of meteoric water interactions

It has been well known that both meteoric and sea water are major ^18^O-depleted natural reservoirs, thus the shift to low δ^18^O values and disequilibrium patterns between zircon and rock-forming minerals in Fig. 1 were attributed to interact with either meteoric or sea water. The following points, however, are inconsistent with seawater interaction: (1) alkali feldspar δ^18^O value down to −1.16‰ with disequilibrium between zircon was observed for the Tianzhushan gneissic country rocks (Table S1 and Fig. 1a), and biotite δ^18^O value of −4.38‰ was measured from the adjacent granitoid pluton^37^,^38^. These low values cannot result from interaction of seawater with δ^18^O value of about 0‰; (2) it can be seen in Fig. S4 that two centers of low δ^18^O meteoric water interactions are outlined for the Tianzhushan granitoid pluton, and its southeastern corner is spatially coincident with the lowered alkali feldspar δ^18^O values for the gneissic country rocks. Given that granitoids experienced meteoric water interactions, the peripheral country rocks should also be infiltrated and convectively circulated inward by the heated meteoric water. In this case, both granitoids and their gneissic country rocks were interacted with the externally infiltrated fluids. The influx of meteoric water not only lowered δ^18^O values but also facilitated to cool down the granitoids. Because of the depth limitation of meteoric water penetration within continents, the observed low alkali feldspar δ^18^O values with steep disequilibrium arrays imply that these granitoids should rapidly emplace high-levels within the heavily eroded and/or highly uplifted gneissic country rocks. Moreover, magmatic water interacted gneiss is more closely distributed around the vicinity of the Tianzhushan granitoid pluton; (3) as shown in Figs. S6 and 3, the lowered δ^18^O values of alkali feldspar and quartz can be well reproduced with modelling of meteoric water interactions; (4) since the seawater is the heaviest endmember with δD value of around 0‰ on the Earth, the seawater related rocks usually have high δD values. However, δD values greater than −40‰ were hardly observed across the Dabie-Sulu orogen^19^,^20^; (5) tectonically, the continent-continent collision between the North and South China Blocks for the Dabie orogen was Triassic^21^,^22^,^23^,^24^,^25^,^26^,^27^,^28^, there was no ocean thereafter leftover to interact with rocks. Therefore, the lowered alkali feldspar and quartz δ^18^O values and disequilibria with zircon observed in this study were ascribed to meteoric water interactions.

Inversion was carried out for sample 01TZS03. Unacceptable initial δ^18^O values of meteoric water (ranging from +5.06 to +10.74‰ for closed- and open-system, respectively), however, were yielded with temperature of the observed quartz-feldspar pair. Given that initial oxygen isotopes of magmatic water were successfully inversed in the preceding section, this probably suggested that the observed temperature could be an apparent instead of reequilibration temperature. Due to the susceptibility of oxygen exchange for alkali feldspar during hydrothermal processes, its δ^18^O value could be somehow elevated by the subsequent water superimposition. Similar scenario occurred for sample 01TZS02.

Two methodologies were taken to inverse the initial δ^18^O values of meteoric water and/or reequilibration temperatures: (1) adjusting the observed δ^18^O values of alkali feldspar to low values; (2) varying the assumed δ^18^O values of meteoric water. The inversed reequilibration temperatures less than 600 °C or initial δ^18^O values of meteoric water less than 0‰ were set as boundary conditions.

It can be seen in Fig. S5 that initial δ^18^O values of meteoric water were inversed when alkali feldspars were reequilibrated with meteoric water at temperatures lower than 400 °C, and systematic low δ^18^O values were yielded for open-system inversions (green dashed vs. solid curves). On the other hand, reequilibration temperatures lower than 400 °C were also inversed with assumed δ^18^O values down to −30‰ for the meteoric water. With the gradual increase of meteoric water δ^18^O values, the inversed reequilibration temperatures were systematically decreased and ultimately converged (blue curves). Actually, the inversed results of the two independent methods above were essentially overlapped with each other, suggesting that they were reliable and reasonable. Because the paleolatitude of the Dabie orogen had almost been fixed since Triassic, the extremely low initial δ^18^O values of meteoric water were less realistic. Moreover, kinetic considerations do not favor lower reequilibration temperatures while it is thermodynamically likely. In this case, the upper limits of reequilibration temperature and corresponding initial δ^18^O value of meteoric water were thus adopted in this study (arrowed black lines in Fig. S5).

As shown in Figs S6 and 3, the observed δ^18^O values of quartz were successfully reproduced for samples 01TZS02 and 01TZS03 with the inversed initial δ^18^O values of meteoric water and reequilibration temperatures (Table S2). W/R ratios slightly over 0.4 were yielded for open-system meteoric water interactions although systematically large W/R ratios were required for closed-systems. The corresponding doD values are −65 ± 1° for both samples (angles between blue dashed and solid curves in Fig. 1).

**Figure 3:**
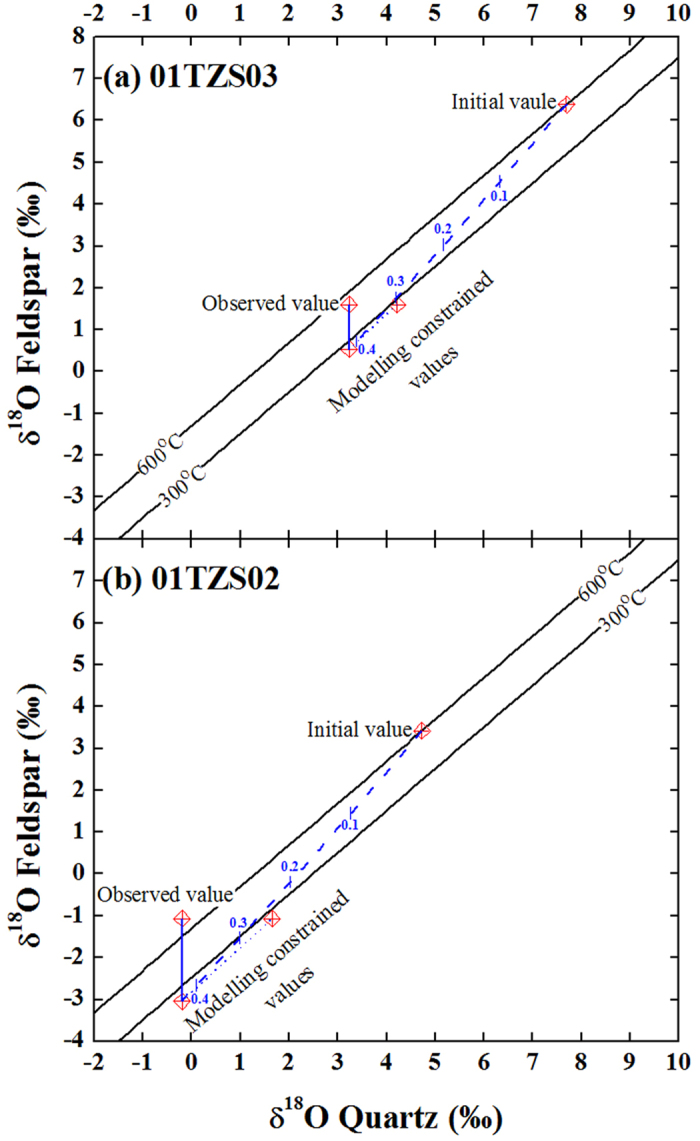
Diagrams of quartz versus alkali feldspar δ^18^O values for the gneissic country rocks interacted with meteoric water in the Dabie orogen. Dashed curves denoted the first-stage interactions of open-system modellings with inversed initial δ^18^O values of meteoric water and corresponding reequilibration temperatures (Table S2). Dotted lines illustrated the second-stage water superimpositions, others refer to Fig. 1.

In order to reproduce the observed δ^18^O values of alkali feldspars, the second-stage water superimpositions were conducted. Setting the final values of the first-stage interactions as initial values for the second-stage water superimpositions, the new reequilibration temperatures were inversed with the observed δ^18^O values of alkali feldspar. They were 280 °C and 265 °C for samples 01TZS03 and 01TZS02 through open-system inversions, respectively. If both quartz and alkali feldspar were reequilibrated with meteoric water during the second-stage water superimpositions, their δ^18^O values should be somehow elevated together (dotted lines in Fig. 3). Due to the sluggish rate of oxygen exchange for quartz, however, only alkali feldspars were actually reequilibrated with meteoric water and their δ^18^O values were preferentially elevated to the observed values (blue solid lines in Fig. 3). In this regard, it is thus the superimposition of two-stage meteoric water interactions that an apparent or disequilibrium pattern between quartz and alkali feldspar oxygen isotopes was yielded for samples 01TZS03 and 01TZS02.

Compared to the modern precipitations^39^, the inversed initial δ^18^O values of meteoric water are somehow low in this study (Fig. S5 and Table S2). These low values probably implied that either the paleoclimate was a little bit cold or the paleoelevation was somehow higher than the present for the Dabie orogen. On the other hand, variable δ^18^O values of meteoric water were inversed for kilometer-apart outcrops. The following points could potentially account for these variabilities: (1) interactions with other rocks prior to the studied samples. As the W/R ratios of meteoric water interactions were less than one in this study (Figs 6S and 3), this geologically suggested that the pervasive convection of meteoric water was lithostatically controlled via porosity within the gneissic country rock. During these processes, the δ^18^O values of circulating meteoric water could be somehow altered through exchange with infiltrated rocks and/or minerals; (2) mixing with high δ^18^O magmatic water. Since the studied gneissic country rocks distributed around the peripheral of the Tianzhushan granitoid pluton (Fig. S4), the binary mixing could also result in δ^18^O variations of inversed meteoric water; (3) age-dependent effect. That is, the inversed meteoric waters were not essentially precipitated at the same time, they probably could be Triassic or Cretaceous meteoric water, respectively. Moreover, as shown in Fig. 3, slightly high reequilibration temperatures were inversed for the second-stage meteoric water superimpositions. These could result from either persistent heating of the adjacent plutonsim or prograde metamorphism. In these respects, the future geochronological datings will play an important role to distinguish them. Nevertheless, some of the Tianzhushan gneissic country rocks were thus overprinted by the externally infiltrated meteoric water.

It is worthwhile pointing out that doD values are more evident for meteoric water interactions (blue dashed curves vs. red one in Fig. 1) whereas their W/R ratios are overall lower than that for magmatic water interactions (Figs S2 and 2 vs. S6 and 3 and Table S3). Because doD values and W/R ratios depended upon the initial oxygen isotopes of constituent minerals and waters, temperatures and even system behaviors, hence a small amount of infiltration of heated meteoric water being far from equilibrium state with constituent minerals could considerably and/or effectively lower the δ^18^O values of alkali feldspar and/or quartz. If the lifetime and/or temperature were long (high) enough for the hydrothermal systems, then reequilibration between alkali feldspar and quartz oxygen isotopes could be achieved. Otherwise, disequilibrium patterns occurred.

### Kinetic consideration and oxygen exchange modelling

Oxygen diffusion modelling showed that both quartz and alkali feldspar can approach reequilibration with water under high-temperature conditions, the time required to reset their original δ^18^O values ranges from less than 30 Kyr to about 0.5 Myr (million years) at 490 °C for sample 01TZS07 (arrowed red dashed lines in Fig. S1b). Since a disequilibrium pattern between quartz and alkali feldspar oxygen isotopes was observed for sample 01TZS07, the duration of internally buffered retrograde metamorphic water interaction should thus be no more than 0.5 Myr.

As listed in Tables S2 and 3, the reequilibration temperatures of externally infiltrated magmatic and meteoric water interactions were less than 350 °C in this study. A minimum time of about 2.5 Myr was required to achieve reequilibration with meteoric water for susceptible alkali feldspar at 250 °C (arrowed blue dashed line in Fig. S1b). Because the rates of oxygen diffusion within quartz are rather low at temperature intervals from 350 to 250 °C (Fig. S1a), the timescale is therefore unreasonably long to reset the quartz δ^18^O values via diffusive oxygen exchange with waters. Given that reequilibrations were achieved and/or reproduced between quartz and alkali feldspar for the studied samples (labelled data points in Figs. 2 and 3), this probably suggested that surface reaction instead of volume diffusion actually controlled the oxygen exchange under low-temperature conditions. Mechanisms such as dissolution, reprecipitation and exchange along micro-fractures and/or within networks were proposed to account for the variation of quartz δ^18^O values during water-rock interactions^40^,^41^,^42^,^43^,^44^.

Based on parameters listed in Table S3, the time required to achieve 99% oxygen exchange between mineral and water was calculated. As the original formulations were proposed for closed-systems^40^, the reequilibration temperatures and W/R ratios inversed from closed-systems were thus adopted in this study. For the magmatic water interacted sample 01TZS05, an upper-limit time of ca. 12 Kyr was required to achieve reequilibration between quartz and alkali feldspar oxygen isotopes (italic bold number with underline in Table S3). In order to reequilibrate with meteoric water for samples 01TZS02 and 01TZS03, a time up to about 36 Kyr was required. For the second-stage meteoric water superimpositions, the timescale less than 2.3 Kyr was inferred to achieve reequilibration only for alkali feldspar but not quartz, and an apparent or disequilibrium pattern between quartz and alkali feldspar oxygen isotopes was accordingly observed (Fig. 3). Therefore, if the magmatic and meteoric water interactions were driven by the postcollisional magmatism in the Dabie orogen, the overall lifetime of fossil hydrothermal systems should not be shorter than 50 Kyr.

### Isotopic evolution of water-rock interactions

The overprint of water interactions on the gneissic country rocks was not only recorded by oxygen isotopes but also unravelled through zircon *in situ* U-Pb datings and cathodoluminescent (CL) imaginings^45^. For example, discordant results were dated for zircon of sample 01TZS05, and its lower intercept age of 126 ± 20 Ma could be attributed to the thermal pulse accompanying with the early Cretaceous magmatic water interaction, which could effectively enhance the diffusive loss of Pb and therefore result in discordance. Its upper intercept of 754 ± 15 Ma pointed to the age of protolith, which petrogenetically related to Neoproterozoic low δ^18^O magma in the South China Block^30^,^31^,^46^,^47^. In contrast, Triassic age of 244 ± 44 Ma was somehow retained for zircon of sample 00DB64 from the Sidaohe, which was not experienced noticeable water interactions and equilibrium oxygen isotope fractionations were well maintained (Fig. 1).

**Figure 4:**
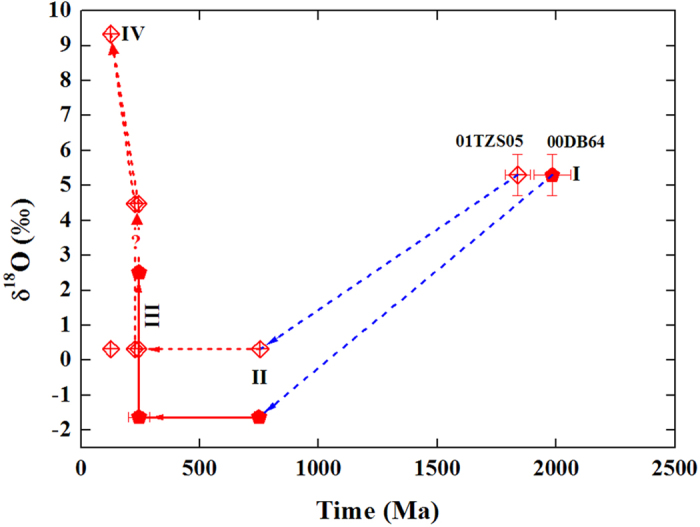
Comparison of isotopic evolutions for the magmatic water interacted gneissic country rock with gneiss unexperienced water interaction. The time of primary magma derived from the crust-mantle differentiation was constrained by zircon Lu-Hf two-stage depleted mantle model age (T_DM2_). Given that the similarity of T_DM2_ and ε_Hf_(T_DM2_) for the two samples, mantle zircon δ^18^O values of 5.3 ± 0.6‰ were thus assumed for the primary magmas. Triassic age prior to the magmatic water overprint for the sample 01TZS05 is after the compilation of metamorphic rocks without coesite in the Dabie orogen^19^,^20^. Note that both zircon and quartz δ^18^O values were illustrated for stages III and IV, and arrowed lines denote hypothetical pathways. Data refer to Table S4.

Combined zircon oxygen isotopes with available U-Pb ages and Lu-Hf isotopic data, four-stage evolutions were proposed for the magmatic water interacted gneissic country rock and summarized as: I. Primary magma with mantle zircon δ^18^O value was derived from crust-mantle differentiation about 1900 Ma; II. Low δ^18^O magma was generated from the Neoproterozoic Rodinia supercontinent rifting magmatism in the South China Block; III. Triassic metamorphism occurred during the continental collision between the North and South China Blocks and equilibrium fractionation of oxygen isotopes between zircon and rock-forming minerals was attained; IV. The early Cretaceous postcollisional magmatic water interaction resulted in disequilibrium pattern of oxygen isotopes between zircon and rock-forming minerals of the intruded gneiss from the Dabie orogen in central-eastern China (Fig. 4).

## Methods

### Analyses of oxygen isotopes

Conventional crushing, gravimetric, heavy liquid and magnetic techniques were applied to separate and concentrate zircon, quartz, and alkali feldspar from whole-rock samples. In order to avoid metamict zircons and other impurities, the separated zircons were sequentially treated with concentrated HCl, HNO_3_ and HF acids under room conditions overnight. The purity of mineral separates is generally better than 98% with optical microscope examination.

Oxygen isotopes were analyzed with the laser fluorination online techniques^48^,^49^, and the conventional δ^18^O notation in permil (‰) relative to Vienna Standard Mean Ocean Water (VSMOW) was reported in Table S1. In order to control the quality of δ^18^O analysis, the garnet standard UWG-2 was routinely analyzed. For 15 analytical days over three months, the daily average of measured δ^18^O values of UWG-2 varied from 5.54 to 5.89‰, and the corresponding analytical precision is better than ±0.11‰ (1 SD). Raw δ^18^O values of mineral separates were accordingly corrected in terms of the accepted UWG-2 value of 5.80‰. The international standard, NBS 28 quartz, was analyzed and the corrected δ^18^O values are from 9.31 to 9.69‰ during the course of this study.

The reproducibility of fresh crystalline zircon δ^18^O analyses is excellent throughout this study. As shown in Table S1, except for one sample, the one standard deviation of most duplicate with two triplicate measurements is less than ±0.10‰, which is within the maximum routine analytical uncertainties demonstrated by daily UWG-2 garnet standard measurements.

### Inversion of water-rock interactions

On the basis of mass balance, water-rock interaction was formulated for forward modelling of oxygen isotopes^2^. For a closed-system, the following relationship is satisfied:


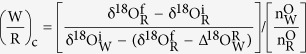


And for an open-system, a simplified relationship is held:


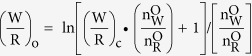


where W/R denoted time integrated water/rock ratio for a closed- or open-system, respectively. δ^18^O values with subscript R or W denoted rock and water, and superscript i and f denoted initial and final oxygen isotopes of rock or water prior to and after water-rock interactions, respectively. 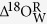 value denoted oxygen isotope fractionation between rock and water, and 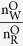 denoted ratio of exchangeable oxygen content between water and rock.

Because partial reequilibrations of oxygen isotopes among rock-forming minerals were achieved during water interactions for some samples in this study (Figs 2 and 3), the initial δ^18^O values of water can be inversed. For a closed-system, two equations were derived for alkali feldspar (Ksp) and quartz (Qtz), respectively:


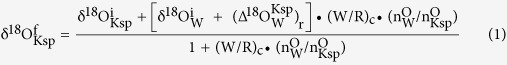



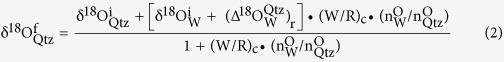



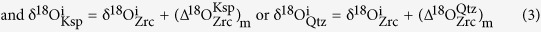


Amongst all of parameters in Eqs. (1) through (3), 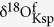 and 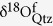 are observed values for a specific sample, 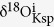 and 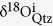 values can be calculated by Eq. (3) with the observed zircon (Zrc) δ^18^O values and metamorphic temperature (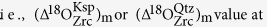 600 °C), and 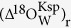
or 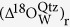
value can be calculated with reequilibration temperature. Moreover, 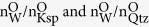 ratios are actually constants between water and alkali feldspar as well as quartz (last row in Table S2). In this case, both 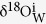 and (W/R)_c_ can thus be uniquely solved by combination of Eqs. (1) and (2).

In order to be self-consistent, theoretically calculated oxygen isotope fractionations were adopted throughout this study^35^. Because the discrepancy between theoretical calculation and experimental calibration or empirical estimation is not remarkable for oxygen isotope fractionations of the studied constituent minerals, this will not considerably influence the results.

For an open-system, a similar inverse procedure can be applied. Due to the term of natural log or exponential function, an analytical expression cannot be obtained. Under this circumstance, a numerical reiteration with precision of at least 0.0001 was conducted to inverse the initial oxygen isotopes of water and reequilibration temperatures and listed in Table S2.

### Degree of disequilibrium (doD)

While kinetic theory was proposed to deal with disequilibrium oxygen isotopes in δ-δ space^50^,^51^, the doD value is alternatively approximated as:


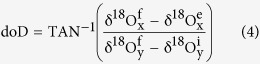


where 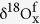 and 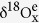 are the observed and thermodynamic equilibrium exchanged δ^18^O values along X-axis (they are referred to zircon in Fig. 1), whereas 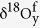 and 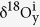 are observed and initial δ^18^O values along Y-axes (they are referred to alkali feldspar and quartz in Fig. 1, respectively). The calculated doD value actually denoted the rotation angle between thermodynamic equilibrium exchanged and observed δ^18^O values of constituent minerals in the course of water-rock interactions.

## Additional Information

**How to cite this article**: Wei, C.-S. and Zhao, Z.-F. Dual sources of water overprinting on the low zircon δ18O metamorphic country rocks: Disequilibrium constrained through inverse modelling of partial reequilibration. *Sci. Rep.* **7**, 40334; doi: 10.1038/srep40334 (2017).

**Publisher's note:** Springer Nature remains neutral with regard to jurisdictional claims in published maps and institutional affiliations.

## Supplementary Material

Supplementary Information
