# Supplementary material for: Dual sources of water overprinting on the low zircon δ18O metamorphic country rocks: Disequilibrium constrained through inverse modelling of partial reequilibration
Source: Sci Rep. 2017 Jan 16;7:40334. doi: 10.1038/srep40334 (PMC5238450; doi:10.1038/srep40334)

Supplementary Information for

Dual sources of water overprinting on the low zircon $\delta^{18}O$ metamorphic country rocks: Disequilibrium constrained through inverse modelling of partial reequilibration

Chun-Sheng Wei & Zi-Fu Zhao

CAS Key Laboratory of Crust-Mantle Materials and Environments, School of Earth and Space Sciences, University of Science and Technology of China, Hefei 230026, CHINA.

Table S1. Oxygen isotopes of gneisses and granitoids from the Dabie orogen, central-eastern China.

Sample	$\delta^{18}O$ Zircon (‰)			$\delta^{18}O$ Quartz (‰)			$\delta^{18}O$ Alkali feldspar (‰)			GPS data
number	Measured	Ave	1SD	Measured	Ave	1SD	Measured	Ave	1SD	
**Sidaohe (SDH)**										
**Gneiss**										
**00DB63**	-0.59, -0.67, -0.64	-0.63	0.04	3.11, 2.91	3.01	0.14	1.77	1.77	/	31º22′12″, 115º04′09″
**00DB64**	-1.75, -1.51	-1.63	0.17	2.53, 2.51	2.52	0.01	1.22	1.22	/	31º22′12″, 115º04′09″
**Tianzhushan/Yuexi pluton (TZS)**										
**Gneiss**										
**01TZS02**	0.69	0.69	/	-0.03, -0.55, -0.02	-0.20	0.30	-0.98, -1.16	-1.07	0.13	30º40′05″, 116º29′07″
**01TZS03**	3.66, 3.67, 3.64	3.66	0.02	3.23	3.23	/	1.60	1.60	/	30º42′04″, 116º23′31″
**01TZS05**	0.33, 0.31	0.32	0.01	9.33	9.33	/	7.06	7.06	/	30º43′24″, 116º26′51″
**01TZS07**	-3.78, -3.71	-3.75	0.05	0.29, 0.14, 0.34	0.26	0.10	-0.26, -0.01	-0.14	0.18	30º42′06″, 116º29′13″

**Granitoid** [a]										
**01TZS06**	5.87, 5.88	5.88	0.01	9.45, 9.26, 9.27	9.33	0.11	5.17	5.17	/	30º42´55″, 116º27´48″
**03TZ01**	4.98	4.98	/	8.14	8.14	/	7.00	7.00	/	30º50´41″, 116º17´14″
**03TZ02**	5.18	5.18	/	8.01	8.01	/	7.08	7.08	/	30º50´31″, 116º18´09″
**03TZ03**	5.57	5.57	/	8.53	8.53	/	5.99	5.99	/	30º50´39″, 116º19´05″
**03TZ05**	5.83	5.83	/	9.16	9.16	/	7.80	7.80	/	30º48´25″, 116º20´45″
**03TZ06**	5.54	5.54	/	/	/	/	3.76	3.76	/	30º46´41″, 116º20´45″
**03TZ08**	5.56	5.56	/	8.50	8.50	/	2.14	2.14	/	30º45´42″, 116º20´34″
**03TZ09**	5.44	5.44	/	8.81	8.81	/	7.40	7.40	/	30º44´20″, 116º22´02″
**03TZ10**	5.14	5.14	/	7.95	7.95	/	6.50	6.50	/	30º43´25″, 116º23´08″
**03TZ11**	5.40	5.40	/	8.33	8.33	/	6.69	6.69	/	30º43´23″, 116º26´52″
**03TZ12**	5.08	5.08	/	7.93	7.93	/	6.27	6.27	/	30º45´22″, 116º26´06″
**03TZ14**	4.16	4.16	/	8.38	8.38	/	6.38	6.38	/	30º44´45″, 116º26´01″

**03TZ16**	4.41	4.41	/	7.48	7.48	/	4.18	4.18	/	30º43´51″, 116º28´02″
**03TZ17**	5.09	5.09	/	7.98	7.98	/	6.44	6.44	/	30º44´26″, 116º27´11″
**03TZ18**	4.94	4.94	/	7.85	7.85	/	6.47	6.47	/	30º44´35″, 116º27´07″
**03TZ19**	5.47	5.47	/	8.28	8.28	/	6.22	6.22	/	30º44´33″, 116º27´27″
**03TZ20**	5.29	5.29	/	8.17	8.17	/	6.10	6.10	/	30º43´56″, 116º27´26″
**03TZ22**	5.44	5.44	/	8.26	8.26	/	7.58	7.58	/	30º44´46″, 116º29´08″
**02TZ01**	5.32	5.32	/	7.90	7.90	/	5.82	5.82	/	30º43´45″, 116º26´49″
**02TZ02**	5.37	5.37	/	8.36	8.36	/	6.70	6.70	/	30º43´40″, 116º26´47″
**02TZ03**	5.17	5.17	/	8.18	8.18	/	6.02	6.02	/	30º43´28″, 116º26´53″
**02TZ04**	5.37	5.37	/	8.26	8.26	/	3.35	3.35	/	30º43´22″, 116º27´19″
**02TZ05**	5.00	5.00	/	7.96	7.96	/	2.32	2.32	/	30º43´38″, 116º27´47″

[a] Except 01TZS06, other samples are from refs. 37, 38.

Table S2. Parameters of oxygen isotopic modelling for open-system water-rock interactions.

Sample number	$\delta^{18}O^{i}_{Zrc}$ (‰) [a]	$\delta^{18}O^{i}_{Qtz}$ (‰) [b]	$\delta^{18}O^{i}_{Ksp}$ (‰) [b]	T (°C)	$\delta^{18}O^{i}_{W}$ (‰)
**01TZS02**	0.69	4.72	3.42	250 [c]	-13.69 [c]
**01TZS03**	3.66	7.69	6.39	270 [c]	-9.25 [c]
**01TZS05**	0.32	4.35	3.05	340 [d]	4.21 [d]
**01TZS07**	-3.75	0.26	-1.02	490 [e]	-0.86 [e]
$n^{O}_{Water}/n^{O}_{Mineral}$ [f]	2.54	1.68	1.93	/	/

[a] Data refer to Table S1.

[b] Calculated with the observed zircon $\delta^{18}O$ values at 600°C, which was retrieved from the sample 01TZS07 and assumed a common thermal regime on kilometer-scale for the gneissic country rocks prior to water-rock interactions.

[c] Inversed constraints refer to Fig. S5 and text.

[d] Inversed constraint with the observed quartz and alkali feldspar $\delta^{18}O$ values.

[e] Inversed constraint with the internally buffered retrograde metamorphic water $\delta^{18}O$ value of -0.86‰, which was calculated with the observed zircon $\delta^{18}O$ values at 600°C.

[f] Ratio of exchangeable oxygen content between water and mineral.

Table S3. Parameters of oxygen exchange modelling for closed-systems.

Sample number	T (°C)	W/R [c]	Quartz					Alkali feldspar				
			Xs [d]	ρ [e] (g/cm^3)	log r [f] (moles O m^{-2} s^{-1})	a [g] (cm)	t [h] (Kyr)	Xs [d]	ρ [e] (g/cm^3)	log r [f] (moles O m^{-2} s^{-1})	a [g] (cm)	t [h] (Kyr)
**01TZS02**	285 [a]	0.86	0.538		-8.65	0.25	***36.0***	0.538		-7.48	0.25	***2.3***
						0.05	7.2				0.05	0.5
**01TZS03**	295 [a]	0.87	0.535	2.66	-8.58	0.25	30.6	0.535	2.56	-7.42	0.25	2.0
						0.05	6.1				0.05	0.4
**01TZS05**	340 [b]	2.85	0.260		-8.27	0.25	***11.6***	0.260		-7.17	0.25	0.9
						0.05	2.3				0.05	0.2

[a] Inversed constraints refer to Fig. S5.

[b] Calculated with the observed quartz and alkali feldspar $\delta^{18}O$ values.

[c] Refer to Figs. S2 and S6.

[d] Mole fraction of mineral oxygen.

[e] Mineral density from ref. 52.

[f] Rate constant after refs. 40, 41.

[g] Grain radius.

[h] Time required to achieve 99% oxygen exchange between mineral and water.

Table S4. Summary of zircon isotopic data [a].

Sample number	U-Pb age		Hf isotopes			$\delta^{18}O$ (‰)
	t_1 (Ma)	t_2 (Ma)	$^{176}Hf/^{177}Hf$	T_{DM2} (Ma)	ε_{Hf} (T_{DM2})	
00DB64	749±14	244±44	0.282185	1991±44	20.9±1.0	-1.63±0.17
01TZS05	754±15	126±20	0.282249	1891±112	20.2±0.5	0.32±0.01

[a] Zircon U-Pb and Hf isotopes are compiled after ref. 45, oxygen isotopes are from this study.

Figure S1. (a) Arrhenius plot of oxygen diffusion in minerals under wet conditions. Dashed lines are theoretical calculations[53], dotted lines with solid segments are experimentally determined. For zircon, WC is from ref. 54 and FG is from ref. 55, respectively. Quartz data are from ref. 56, and orthoclase is after ref. 57. **(b) Oxygen diffusion modelling for quartz with two radii (solid line based on experimentally determined parameters, and dashed line on theoretical ones) and orthoclase.** Model of spherical crystals of $Dt/a^2=0.03$ was adopted, and arrowed lines denote the timescale of meteoric (blue) and retrograde metamorphic (red) water interactions of open-systems, respectively.

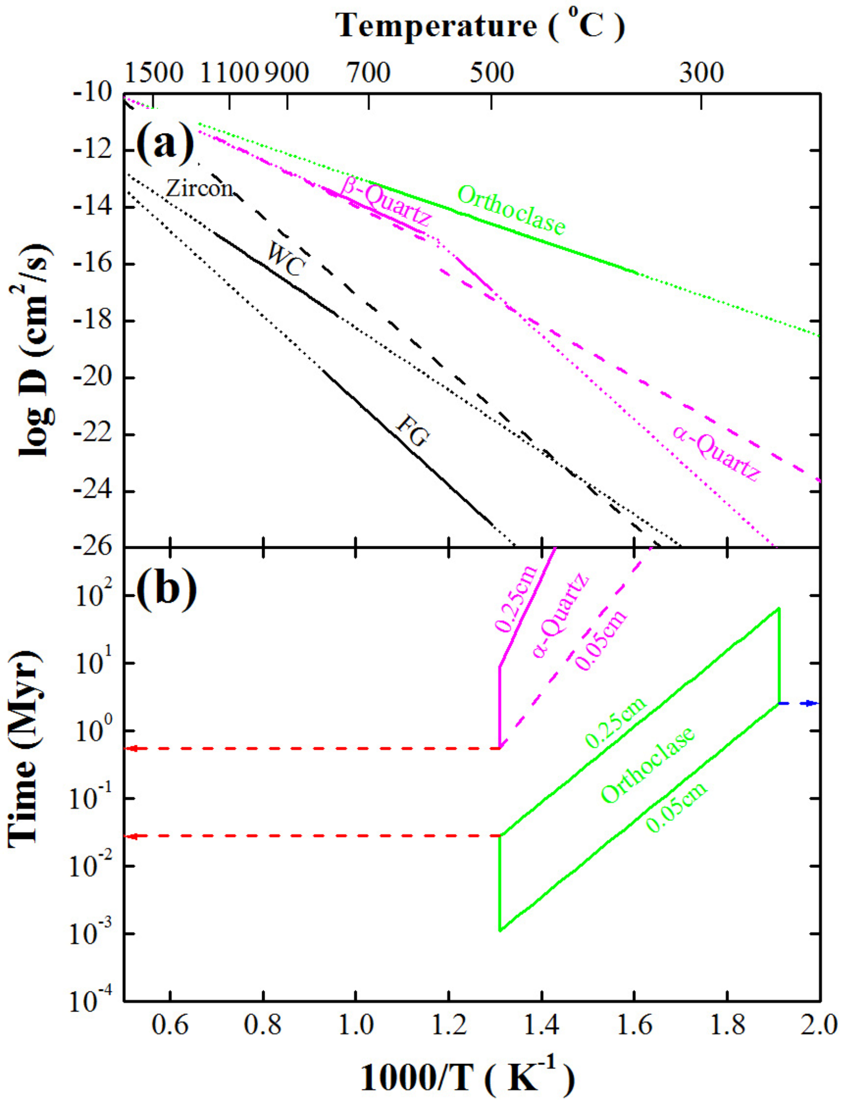

Figure S2. Modelling of high $\delta^{18}O$ water interactions for the observed alkali feldspar (a) quartz (b) and (c) zircon $\delta^{18}O$ values of samples 01TZS05 and 01TZS07, respectively. Arrowed vertical lines in (a) and (b) denoted W/R ratios, and data and parameters refer to Tables S1 and S2.

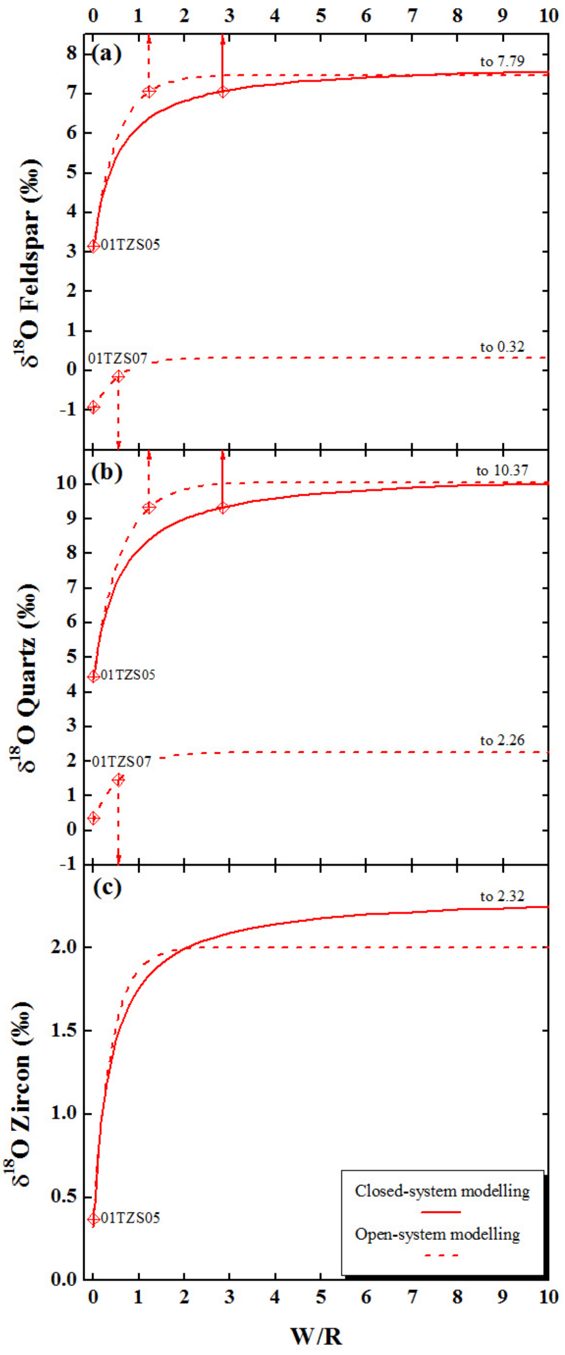

Figure S3. Hypothetical evolutions of magmatic water. A red symbol point with error bars denoted the average Teq value of 775±35°C calculated with quartz-zircon pairs and $\delta^{18}O$ value of magmatic water (7.99±0.40 calculated with zircon $\delta^{18}O$ value and Teq) derived from the Tianzhushan granitoid pluton (data refer to Table S1). The blue symbol point is inversed initial $\delta^{18}O$ value of meteoric water from sample 01TZS02, and the black symbol point denoted magmatic water required to reproduce the observed values. Line 1 denoted the diabatic cooling of magmatic water to $\delta^{18}O$ value of 4.21‰, a low temperature around 175°C was resulted in this case. Line 2-1 denoted a mixture with $\delta^{18}O$ value of 4.21‰ and temperature of 340°C required to model magmatic water interactions, in this case an unacceptably low temperature of about -1720°C was resulted for the involved meteoric water. Line 2-2 denoted direct mixing between magmatic and meteoric water with a temperature of 25°C, either an anomalously low $\delta^{18}O$ value of about -4.58‰ or a high temperature of about 645°C was yielded for the mixture. Line 2-3 denoted an isothermal binary mixing between cooled magmatic and heated meteoric water under condition of 340°C, the involvement of less than 20% meteoric water resulted in $\delta^{18}O$ value of 4.21‰ for the mixed magmatic water. It is worthwhile pointing out that if inversed initial $\delta^{18}O$ value of meteoric water from sample 01TZS03 was adopted, a slightly high involvement of meteoric water was required.

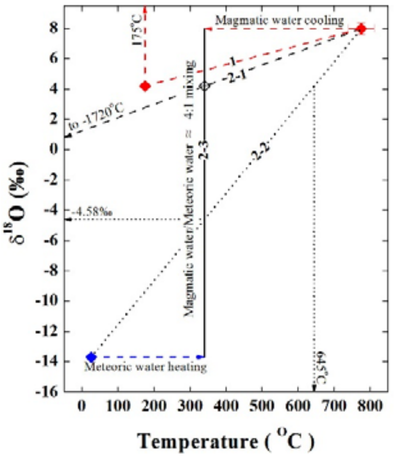

Figure S4. Contour map of the alkali feldspar δ¹⁸O values of the Tianzhushan granitoid pluton in the DBB. Labelled data points are gneissic country rocks for comparison, and red and blue italic bold data within brackets denoted alkali feldspar δ¹⁸O values experienced magmatic (or retrograde metamorphic) and meteoric water interactions, respectively. Data refer to Table S1.

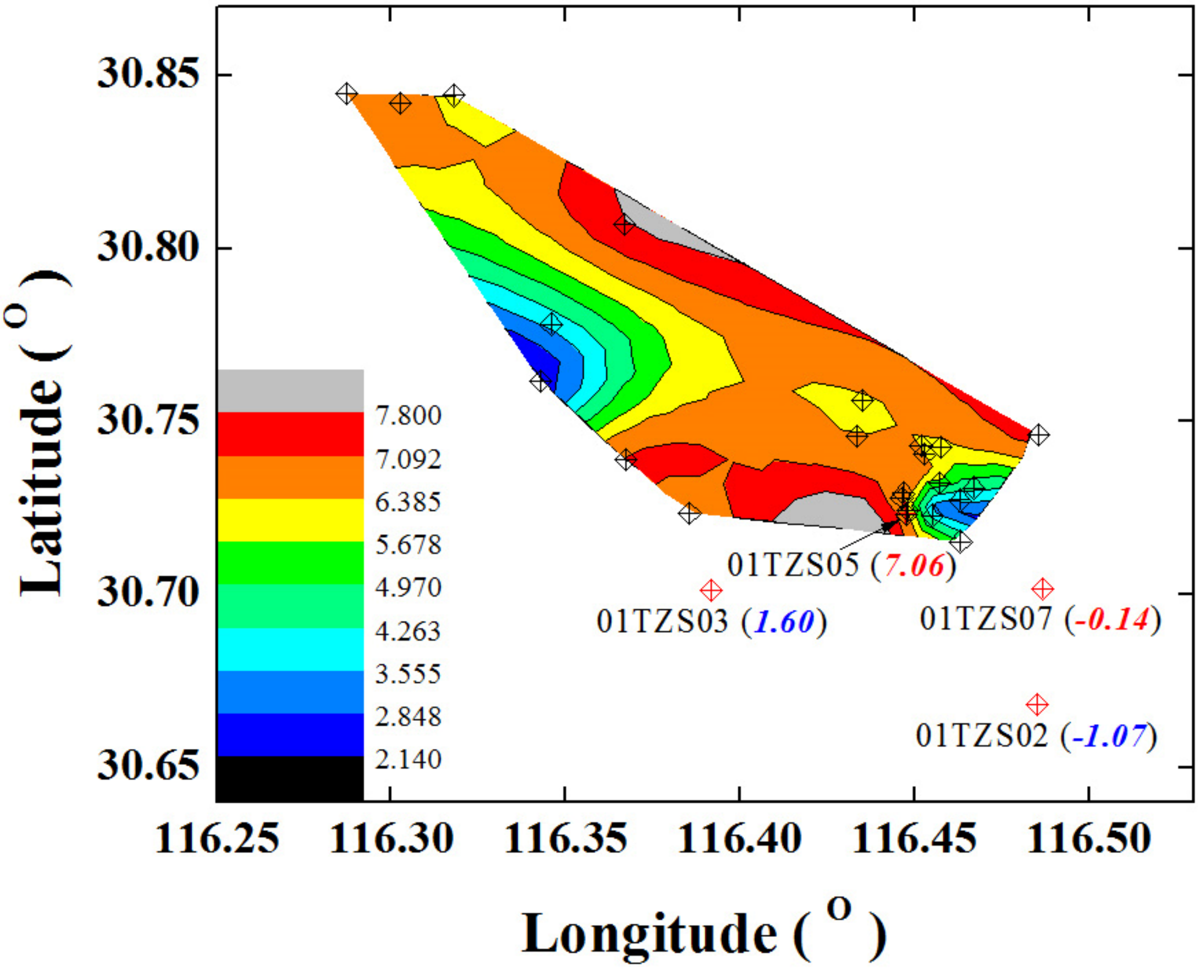

Figure S5. Inversed initial $\delta^{18}O$ values and temperatures reequilibrated with meteoric water for sample 01TZS03 (a) and 01TZS02 (b), respectively. Green curves were inversed with adjustment of alkali feldspar oxygen isotopes to low $\delta^{18}O$ values whereas blue curves were yielded with varied $\delta^{18}O$ values of meteoric water. Arrowed lines denoted upper limits of initial $\delta^{18}O$ value and temperature reequilibrated with meteoric water, respectively, and horizontal lines illustrated modern precipitation[39].

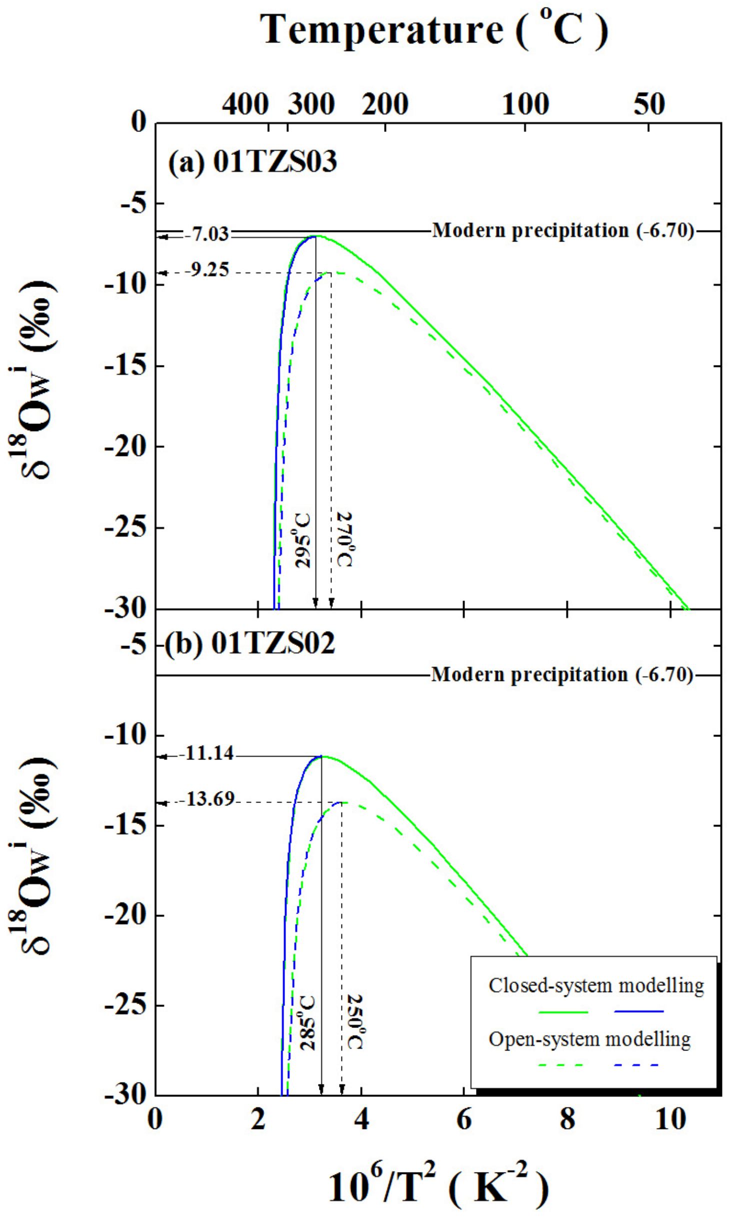

Figure S6. Modelling of meteoric water interactions for alkali feldspar (a) quartz (b) and (c) zircon $\delta^{18}O$ values of samples 01TZS03 and 01TZS02, respectively. Arrowed vertical lines in (a) and (b) denoted W/R ratios with inversed initial $\delta^{18}O$ values of meteoric water and reequilibration temperatures. Note that log scales were adopted for X axes for clarity. Details refer to Table S2 and discussions in text.

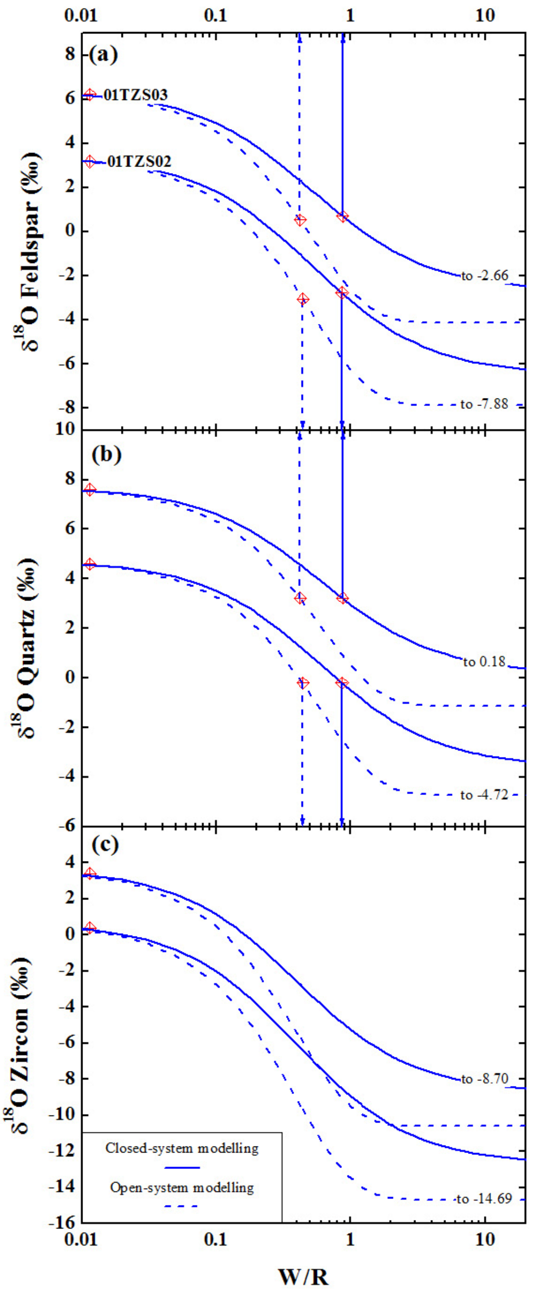

Supplement: Supplementary Information [file srep40334-s1.pdf]
